# Identification and functional analysis of growth- regulating factors involved in abiotic stress response in *Chrysanthemum* L.

**DOI:** 10.3389/fpls.2025.1744200

**Published:** 2026-01-20

**Authors:** Ye Xu, Jingjing Li, Kun Liu, Junping Zheng, Kai Li, Huihui Wang, Hongtao Liu

**Affiliations:** 1 Key Laboratory of Chinese Medicinal Resource and Chinese Herbal Compound of the Ministry of Education, College of Basic Medical Sciences, Hubei University of Chinese Medicine, Wuhan, Hubei, China; 2 Hubei Shizhen Laboratory, Wuhan, Hubei, China; 3 School of Laboratory Medicine, Hubei University of Chinese Medicine, Wuhan, Hubei, China

**Keywords:** abiotic stress, *Chrysanthemum*, gene structure, GRF, phylogenetic analysis

## Abstract

**Introduction:**

*Chrysanthemum* (*Chrysanthemum* L.) is highly valued for its ornamental appeal and medicinal properties. However, its growth is often adversely affected by various abiotic stresses. Growth-regulating factors (GRFs) are a class of plant-specific transcription factors, functioning critically in regulating development, growth processes, hormonal pathways, and environmental stress adaptation. However, the GRF gene family in *Chrysanthemum* species remains poorly characterized.

**Methods:**

GRF genes were systematically identified from four *Chrysanthemum* species (*C. indicum*, *C. makinoi*, *C. nankingense*, and *C. lavandulifolium*), followed by phylogenetic, evolutionary, and expression analyses. Subcellular localization and transcriptional activation assays were performed to characterize key GRF members.

**Results and Discussion:**

A total of 43 GRF genes were identified. Phylogenetic and evolutionary analyses revealed that these genes can be classified into four major clades, with their expansion primarily driven by whole-genome duplication and segmental duplication events. Expression profiling under various abiotic stress conditions - including NaCl, CdCl_2_, drought, high temperature, and low temperature - showed that many GRFs were significantly upregulated. Notably, *CiGRF7* exhibited an expression increase of over 1000-fold under cold and CdCl2 stress, suggesting its key role in abiotic stress responses in *Chrysanthemum*. Subcellular localization confirmed the nuclear localization of the CiGRF7 protein. Additionally, transcriptional activation assays in yeast demonstrated that CiGRF7 possesses self-activating capabilities. Overall, this study provides a comprehensive overview of the GRF gene family in *Chrysanthemum*, shedding light on their evolutionary patterns and stress-responsive expression, and offers valuable genetic resources for future research on stress tolerance and growth regulation in this important ornamental and medicinal plant.

## 1 Introduction

Growth-regulating factors (GRFs) are a class of plant-specific transcription factors that are widely distributed across mosses, ferns, gymnosperms, and angiosperms (Omidbakhshfard et al., 2015; Hu et al., 2023). GRF family proteins typically contain two highly conserved domains: the QLQ (QX3LX2Q) and WRC (CX9CX10CX2H) motifs (Zhang et al., 2008). The QLQ domain, initially identified in the yeast SWI2/SNF2 (SWITCH2/SUCROSE NONFERMENTING2) ATPase complex, is involved in chromatin remodeling by mediating protein - protein interactions. It enables GRFs to interact with GRF-interacting factors (GIFs) through the conserved SNH domain, thereby activating downstream transcription (Kuijt et al., 2014). The plant - specific WRC domain contains a nuclear localization signal (NLS) and a zinc finger motif, enabling DNA binding and nuclear targeting of transcription factors to regulate target gene expression (Kim and Tsukaya, 2015; Omidbakhshfard et al., 2015; Wang et al., 2023a).

Extensive research has established that GRFs play pivotal roles in plant growth and development. For instance, *OsGRF1*, the first identified GRF member, is induced by gibberellin (GA) and promotes leaf and stem growth in rice (van der Knaap et al., 2000; Lu et al., 2020). In *Arabidopsis*, *AtGRF4*/*5*/*6* negatively regulate the expression of *KNOTTED-LIKE FROM ARABIDOPSIS THALIANA2* (*KNAT2*), thereby modulating root meristem development (Kim and Lee, 2006; Kuijt et al., 2014; Hu et al., 2023). In maize, *ZmGRF1* and *ZmGRF10* promote and inhibit cell proliferation, respectively, affecting leaf size in a tissue-specific manner (Wu et al., 2014; Nelissen et al., 2015). Several GRF genes, such as *OsGRF4*, *OsGRF7*, and *OsGRF8*, have been shown to increase grain size and improve crop yield through the regulation of hormone biosynthesis. Additionally, *OsGRF6* enhances the development of secondary branches by activating the *OsYUCCA1*-mediated auxin signaling pathway, contributing to yield improvement in rice (Gao et al., 2015; Yuan et al., 2024a). In *Brassica napus*, high expression of GRF genes correlates with increased yield traits, and heterologous expression of *BnGRF2a* significantly enhances leaf size and oil content (Liu et al., 2012; Wang et al., 2023a).

GRFs act as key regulators in plant responses to biotic and abiotic stresses. *AtGRF1* and *AtGRF3* modulate the growth-defense balance, enhancing nematode resistance through root cell reprogramming (Hewezi et al., 2012). For abiotic stress, *AtGRF7* negatively regulates ABA signaling and modulates salt/drought responses. The *grf7* mutant exhibits markedly enhanced stress tolerance (Kim et al., 2012). In rice, *OsGRF6* activates salt tolerance pathways by binding to the promoter of *MYB3R*, increasing survival rates of transgenic plants by 74.4% (Yuan et al., 2024b). *OsGRF7* contributes to arbutin biosynthesis and salt tolerance by regulating UDP-glucosyltransferase genes (*OsUGT1/5*) (Chen et al., 2024), while the *OsmiR396-OsGRF8* module improves brown planthopper resistance via flavonoid biosynthesis by activating *OsF3H* (Dai et al., 2019).

*Chrysanthemum* (*Chrysanthemum* L.), a traditional Chinese ornamental, possesses significant medicinal value for functional foods and anti-inflammatory agents (Shinoyama et al., 2012; Wang et al., 2014b; Song et al., 2018). However, environmental stresses from climate change (drought, salinity, heat) reduce *Chrysanthemum* biomass and secondary metabolite homeostasis (Sahithi et al., 2021; Zhou et al., 2021, 2025). Thus, identifying key regulatory genes enhancing stress resilience is essential. GRFs mediate plant growth through cell proliferation and stress responses (Omidbakhshfard et al., 2015; Zhang et al., 2018a; Kim, 2019). Although well-characterized in *Arabidopsis*, rice, and maize, systematic studies on *Chrysanthemum* GRFs remain scarce, particularly regarding abiotic stress functions.

To address this gap, we conducted a systematic analysis of GRF genes in four *Chrysanthemum* species: *C. indicum*, *C. makinoi*, *C. nankingense*, and *C. lavandulifolium*. We characterized their gene structures, expression profiles, physicochemical properties, and *Cis*-regulatory elements (CREs). Results indicate that GRFs play important roles in mediating abiotic stress responses in *Chrysanthemum*. This study provides foundational insights into the molecular mechanisms of stress tolerance and broadens the understanding of GRF gene family functional diversity.

## 2 Materials and methods

### 2.1 Identification of GRF gene family members

To identify GRF genes in *Chrysanthemum* species, we analyzed genomes and protein sequences of *C. indicum*, *C. makinoi*, *C. nankingense*, and *C. lavandulifolium* (https://cgd.njau.edu.cn/). Conserved domain HMMs (WRC: PF08879; QLQ: PF08880) were retrieved from Pfam (http://pfam.xfam.org/) to query genomes via HMMER 3.0 and BLASTp. After removing redundancies, the candidate sequences were validated using SMART (https://smart.embl.de/) to confirm the presence of WRC/QLQ domains. Verified proteins were designated as GRF members.

### 2.2 Gene structure annotation, motif analysis, and chromosomal localization

Using General Feature Format (GFF) files, we extracted GRF gene chromosomal locations containing intron, exon, and noncoding UTR data. Gene structures were visualized via TBtools and R packages (Chen et al., 2023). Conserved motifs were identified via MEME (http://meme-suite.org/tools/meme; max motifs = 20) (Wang et al., 2023a).

### 2.3 Phylogenetic tree construction and collinearity analysis

GRF protein alignments used ClustalW, phylogenetic trees were constructed via the Neighbor-Joining (NJ) method (MEGA 7.0, 1000 bootstraps), and collinearity was analyzed with MCScanX and visualized via Circos (Wang et al., 2023a, 2024).

### 2.4 Physicochemical properties and structural prediction of GRF proteins

GRF protein physicochemical properties - amino acid count, molecular weight, theoretical pI, and GRAVY - were calculated via ProtParam (https://web.expasy.org/protparam/). Secondary structures (α-helices, β-turns, random coils, extended strands) were predicted using SOPMA. Tertiary structures were modeled with SWISS-MODEL (https://swissmodel.expasy.org/interactive) and visualized in PyMOL (Cheng et al., 2023).

### 2.5 Selection pressure analysis of GRF genes

Selection pressure was assessed by calculating synonymous (Ks) and nonsynonymous (Ka) substitution rates plus Ka/Ks ratios using TBtools. Ratios >1 denote positive selection, = 1 neutral evolution, and < 1 purifying selection-reflecting evolutionary conservation. Higher |Ka/Ks| values indicate stronger selection pressure (Hurst, 2002).

### 2.6 CREs prediction

The 2,000 bp upstream promoter regions of GRF genes were extracted and submitted to the PlantCARE database (http://bioinformatics.psb.ugent.be/webtools/plantcare/html/) for CREs prediction and functional annotation. CREs were categorized into three groups: growth and development, hormone responsiveness, and stress responsiveness.

### 2.7 RNA extraction and quantitative real-time PCR analysis

Total RNA was extracted from *C. indicum* leaves using the TaKaRa Plant RNA Extraction Kit. First-strand cDNA was synthesized using the HiScript III First Strand cDNA Synthesis Kit with gDNA Wiper (Vazyme, Nanjing, China). qRT-PCR was conducted using the Applied Biosystems™ platform and 2× FastHS SYBR QPCR Mixture (Allmeek, Beijing, China). CiEF1α was used as the internal reference gene, and relative expression levels were calculated using the 2^-ΔΔCT^ method.

### 2.8 Plant materials and stress treatments

*C. indicum* was selected as the experimental material to examine the expression patterns of GRF genes under various abiotic stresses. Five stress treatments were administered: salt stress (NaCl), heavy metal stress (CdCl_2_), drought stress, cold stress (4°C), and heat stress (45°C). Leaf samples were collected at 0, 3, 6, 9, 12, 24, and 48 h following the onset of stress treatments. Immediately after collection, samples were flash-frozen in liquid nitrogen and stored at -80°C to maintain RNA integrity. Before stress application, plants were cultivated in a controlled growth chamber at 28°C with a 14 h light/10 h dark photoperiod for 60 days.

### 2.9 Subcellular localization

The coding sequences (CDSs) of *CiGRF7* were amplified from *C. indicum* cDNA and cloned into the pBin-GFP vector to generate 35S::*CiGRF7*-sGFP constructs. These constructs, along with the empty vector, were introduced into *Nicotiana benthamiana* leaves via *Agrobacterium tumefaciens* strain GV3101. GFP fluorescence signals were observed using an FV1000 confocal laser scanning microscope to determine subcellular localization.

### 2.10 Transcriptional activation assay

To examine the transcriptional activation activity of *CiGRF7*, their CDSs were cloned into the pGBKT7 and pGADT7 vectors to generate fusion constructs (Fan et al., 2023). These were transformed into yeast strain AH109 using the PEG/LiAc method (Weidi Biotechnology, Shanghai, China). Transformants were plated on SD/-Trp-Leu and SD/-Trp-Leu-His-Ade media and incubated at 30°C for 3–5 days to assess growth and transcriptional activity.

### 2.11 Statistical analysis

All experiments were conducted with three biological replicates. Statistical analyses were performed using GraphPad Prism 9. Student’s *t*-tests were used to assess significance levels: *P* < 0.05 (*), *P <* 0.01 (**), and *P <* 0.001 (***). All qRT-PCR primer sequences used in this study are listed in **Supplementary Table S1**.

## 3 Results

### 3.1 Identification and characterization of the GRF gene family in *Chrysanthemum* species

**Figure 1:**
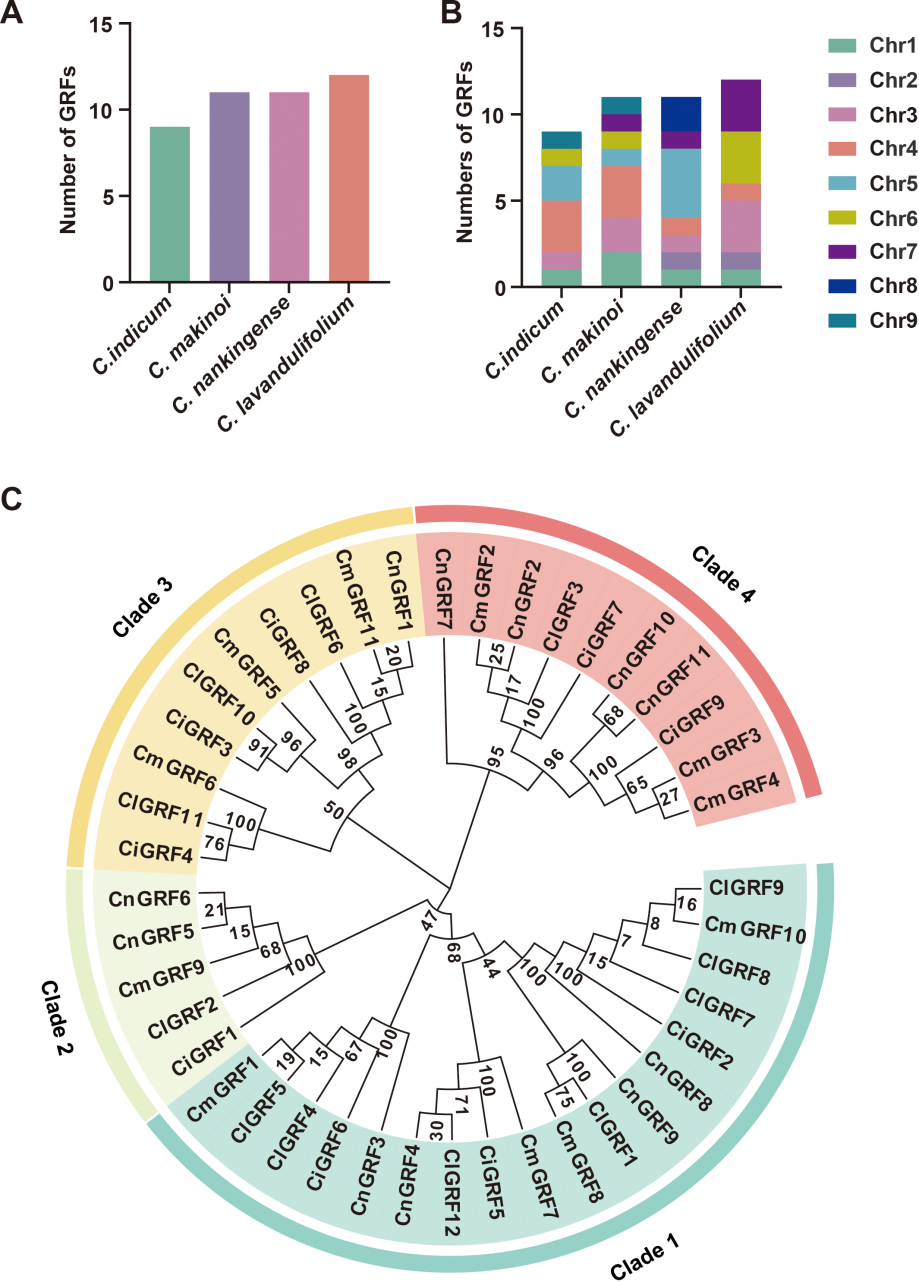
Identification and phylogenetic analysis of GRF genes and proteins in four *Chrysanthemum* species (*C. indicum*, *C. makinoi*, *C. nankingense*, and *C. lavandulifolium*). **(A)** Numbers of GRF genes identified in the four *Chrysanthemum* species; **(B)** Chromosomal distribution of GRF genes in each species; **(C)** Phylogenetic tree of GRF protein sequences with colors denote subfamily classification (Clades 1-4).

To comprehensively identify GRF genes in *Chrysanthemum* species, both BLASTp and HMMER searches were performed using conserved domains WRC (Pfam: PF08879, InterPro: IPR014977) and QLQ (Pfam: PF08880, InterPro: IPR014978) domains. A total of 43 GRF genes were identified across *C. indicum*, *C. makinoi*, *C. nankingense*, and *C. lavandulifolium* (**Figure 1A**, **Supplementary Table S2**), including 9 in *C. indicum*, 11 in *C. makinoi*, 11 in *C. nankingense*, and 12 in *C. lavandulifolium*, indicating relatively stable gene family size across species (**Figure 1A**). The chromosomal distribution was uneven, with GRF genes mostly enriched on chromosomes 4 and 5 (**Figure 1B**), while chromosomes 2, 8, and 9 harbored only two. Furthermore, interspecies variation was observed; for example, GRFs in *C. makinoi* were predominantly located on chromosome 4, whereas those in *C. nankingense* were clustered on chromosome 5 (**Figure 1B**).

Analysis of the physicochemical properties revealed substantial diversity among GRF proteins, with amino acid lengths ranging from 146 (CiGRF1, CmGRF9, CnGRF5, and CnGRF6) to 501 (CmGRF6 and ClGRF11), molecular weights from 16.31 to 54.91 kDa, and theoretical isoelectric points (pI) between 6.17 and 9.57 (**Supplementary Table S2**). To further explore the evolutionary relationships, a phylogenetic tree was constructed using MEGA 7.0, grouping the 43 GRF proteins into four major clades (clade 1 - 4). GRF proteins from the four *Chrysanthemum* species were found in all clades. Clade 1 was the largest group (41.86%), followed by clade 3 and clade 4 (23.26%), while clade 2 contained the fewest members (**Figure 1C**).

### 3.2 Gene structure and motif analysis of GRFs

**Figure 2:**
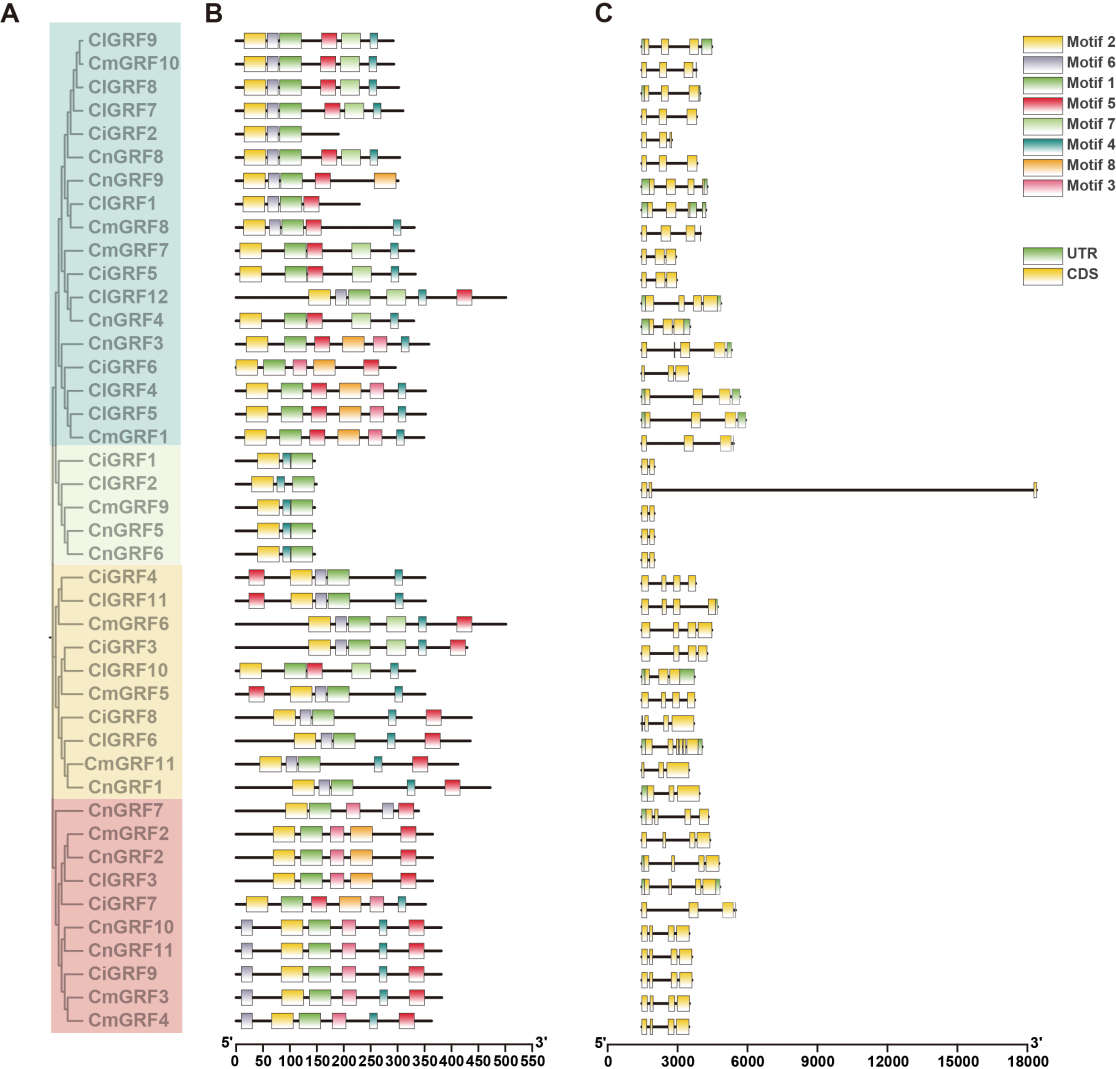
Conserved motifs and gene structure of GRF genes in *Chrysanthemum* species. **(A)** Phylogenetic tree of GRF proteins constructed with MEGA based on protein sequences; **(B)** Distribution of conserved motifs, with colored boxes representing distinct motifs; **(C)** Exon-intron structures of GRF genes in four *Chrysanthemum* species.

To examine structural diversity and functional implications of GRF genes, we analyzed exon-intron structures and conserved motifs across all family members (**Figure 2**). MEME identified eight conserved motifs. Motif 1 and Motif 2 occurred in most GRFs, suggesting essential roles in structural - functional conservation. Members within the same clade exhibited similar motif compositions; clade 2 genes typically harbored only two/three exons and fewer motifs, reflecting simplified structures (**Figures 2A, B**). Additionally, exon numbers ranged from 2 - 6, with > 70% genes harboring 3–4 exons, indicating relatively conserved gene architecture (**Figure 2C**).

Secondary structure prediction revealed GRF proteins were dominated by random coils (67.79% -87.40%), followed by α-helices (8.01% - 22.82%) and extended strands (1.31% - 9.11%), with β-turns least abundant (**Supplementary Table S3**). Three-dimensional models of GRF proteins were generated using SWISS-MODEL and are shown in **Supplementary Figures S1-S4**. Although the GRFs differed in overall length and morphology, all modeled proteins contained the conserved QLQ and WRC domains, which are characteristic features of plant GRF proteins. Structural modeling predicted that the QLQ domain is mainly composed of two consecutive α-helices, whereas the WRC domain lacks well-defined secondary structural elements and appears relatively flexible, suggesting a conserved structural organization of these domains across the GRF family.

### 3.3 Synteny and duplication analysis of GRF genes

**Figure 3:**
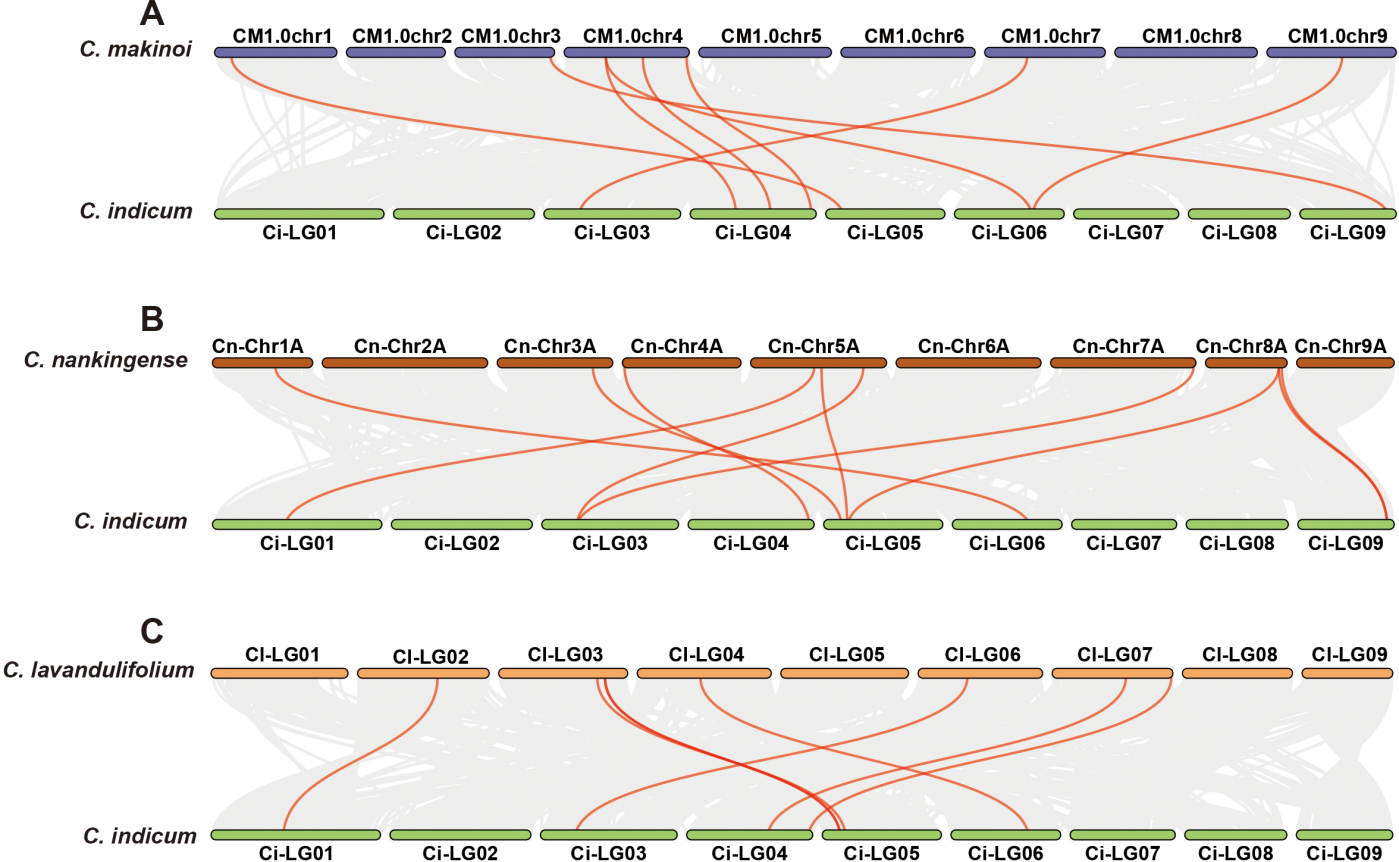
Syntenic relationships of GRF genes between *C. indicum* and other species. **(A)***C. makinoi*; **(B)***C. nankingense*; **(C)***C. lavandulifolium*.

To investigate the evolutionary conservation and expansion of GRF genes in *Chrysanthemum* species, collinearity analysis was performed between *C. indicum* and *C. makinoi*, *C. nankingense*, and *C. lavandulifolium* using MCScanX. The analysis revealed 8, 10, and 7 collinear pairs, respectively (**Figure 3**). The strongest syntenic relationship was observed between *C. indicum* and *C. nankingense*, suggesting that *C. nankingense* is evolutionarily closely related to *C. indicum* and has retained a higher number of ancestral genes.

**Figure 4:**
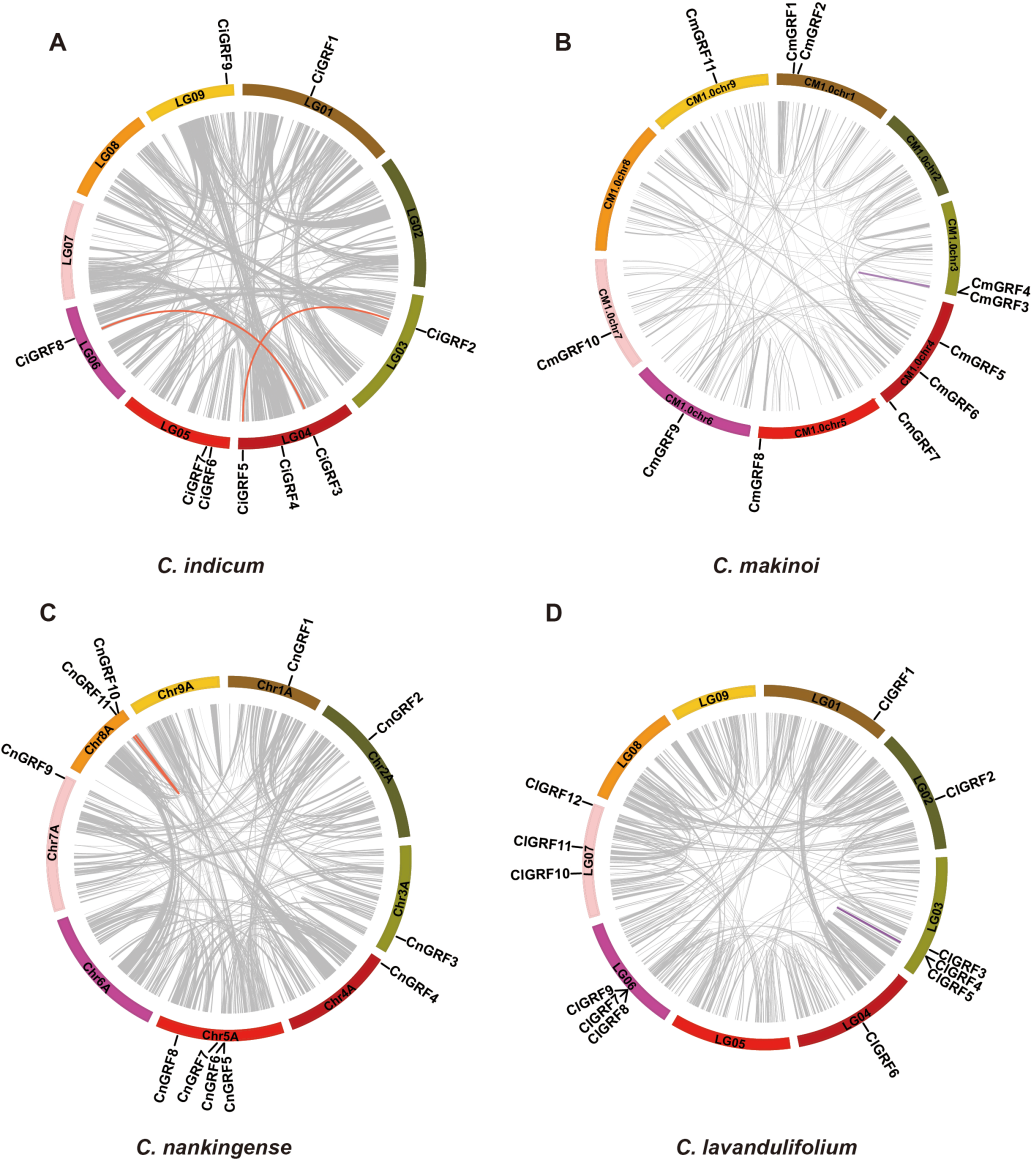
Chromosomal distribution and duplication relationships of GRF genes in four *Chrysanthemum* species. **(A)***C. indicum;***(B)***C. makinoi*; **(C)***C. nankingense;***(D)***C. lavandulifolium*. The positions of GRF genes were annotated using Circos software, and gene pairs resulting from whole-genome duplication (WGD) or segmental duplication were connected with red and purple lines, respectively.

Further analysis identified 5 segmental duplication events across the four species, with *C. indicum* exhibiting the highest frequency (two pairs), while only one duplication events were detected in each of the other *Chrysanthemum* species (**Figure 4**). This implies greater GRF gene stability in *Chrysanthemum* species. Ka/Ks analysis revealed all duplicated pairs had ratios <1 (**Supplementary Table S4**), indicating they had undergone purifying selection during evolution with strongly conserved GRF gene structure/function (Wang et al., 2024).

### 3.4 Spatiotemporal expression analysis of GRF genes

**Figure 5:**
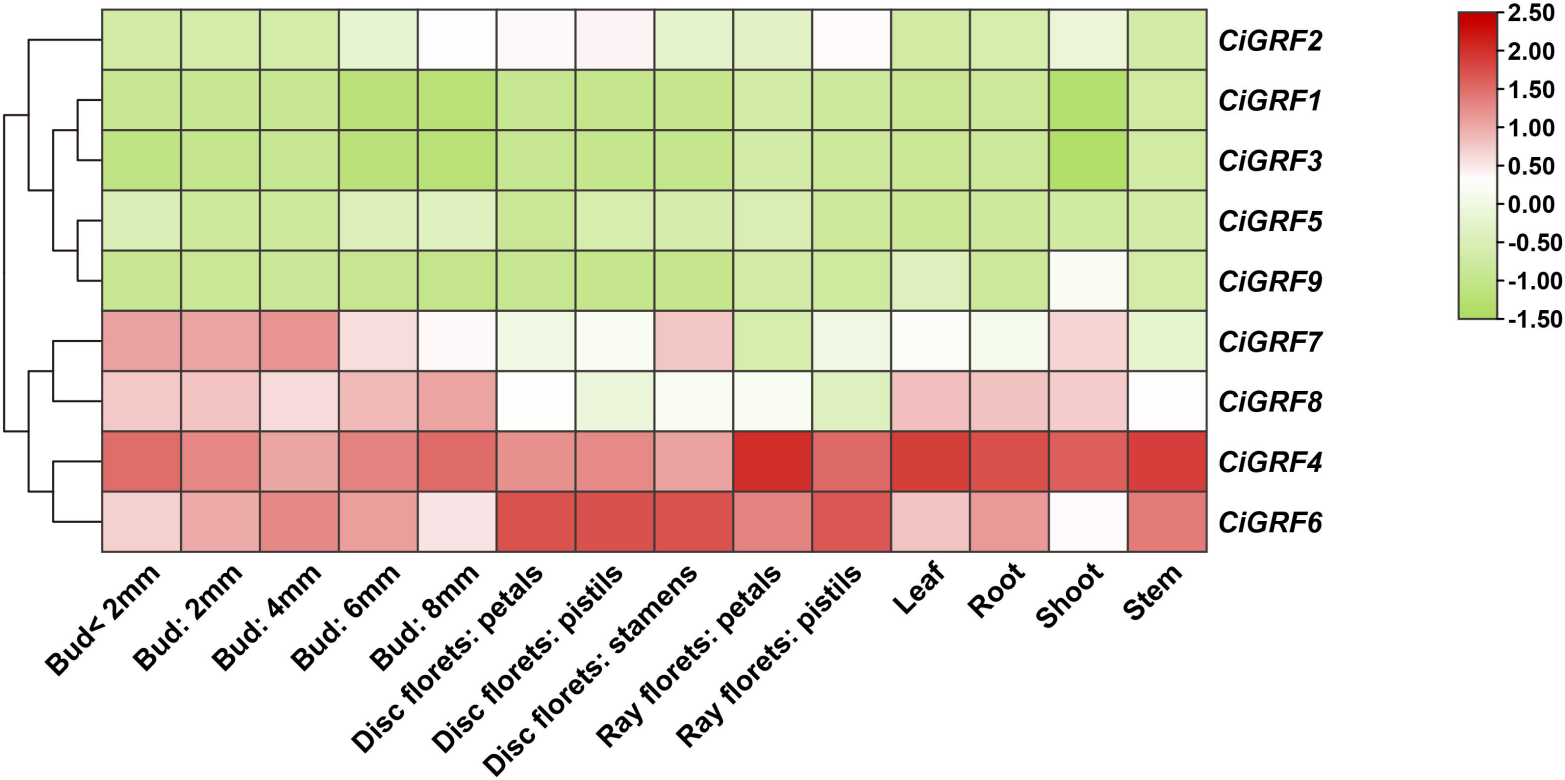
Expression patterns of GRF genes in different tissues of *C. indicum* at various developmental stages. 14 samples were used for expression analysis, including roots, stems, buds (< 2 mm, 2 mm, 4 mm, 6 mm, and 8 mm), leaves, shoots, ray florets, and disc florets.

Previous studies have shown that GRF genes regulate multiple processes in plant growth and development. To explore their potential functions in *Chrysanthemum*, we analyzed the expression profiles of nine *CiGRFs* using publicly available transcriptomic data (https://cgd.njau.edu.cn/). Expression was evaluated in different tissues - roots, stems, buds, leaves, shoots, ray florets, and disc florets - as well as in floral buds at five developmental stages (< 2 mm, 2 mm, 4 mm, 6 mm, and 8 mm) (**Figure 5**).

The results showed that most *CiGRFs*, including *CiGRF4*, *CiGRF6*, *CiGRF7*, and *CiGRF8*, were significantly upregulated during floral bud development, suggesting their involvement in flower organogenesis (**Figure 5**). Additionally, *CiGRF4* and *CiGRF6* exhibited relatively high expression in vegetative organs, such as roots, stems, buds, and leaves, implying multiple roles in both vegetative and reproductive development (**Figure 5**). In contrast, *CiGRF1*, *CiGRF3*, *CiGRF5*, and *CiGRF9* displayed consistently low expression across all tissues, indicating possible tissue specificity or temporal regulation (**Figure 5**). Overall, the expression profiles of *CiGRF* genes varied across organs and developmental stages, highlighting their functional diversity and complexity.

### 3.5 CREs analysis of GRF gene promoters

CREs, which are key transcription factor binding sites within promoter regions, play important roles in regulating gene expression and environmental adaptation (Cao et al., 2024). To investigate the regulatory potential of *GRF* genes, 2,000 bp upstream promoter sequences of each gene from four *Chrysanthemum* species were extracted and analyzed using the PlantCARE database.

**Figure 6:**
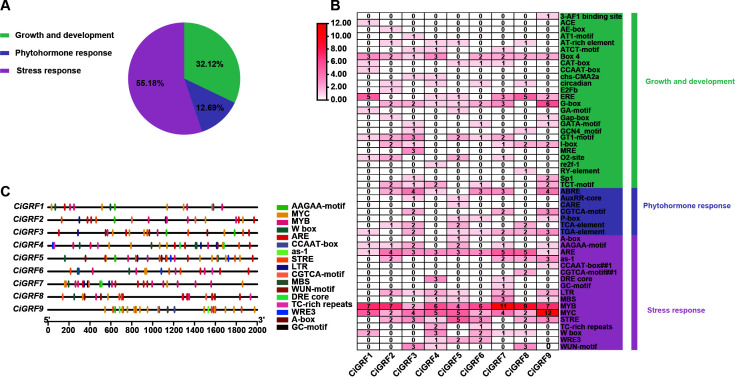
Analysis and identification of CREs in GRFs of *C. indicum*. **(A)** The proportion of CREs in three categories (growth and development, phytohormone response, and stress response). **(B)** Distribution of CREs of GRF genes in different categories. **(C)** Distribution of major stress-related CREs in the promoter sequences of the GRF genes from *C. indicum*.

In *C. indicum*, a total of 50 types of CREs were identified in *Ci*GRF gene promoters, including elements related to meristem development, light signaling, cold response, gibberellin (GA), methyl jasmonate (MeJA), and auxin. Based on functional annotation, the elements were classified into three main categories: growth and development, phytohormone response, and stress response (**Figure 6**). Among them, stress-responsive elements accounted for 55.18%, which is significantly higher than development-related (32.12%) and phytohormone-responsive elements (12.69%) (**Figure 6A**). Notably, MYB- and MYC-binding sites, which are known to mediate responses to abiotic stress, were widely distributed across *CiGRF* promoters (**Figures 6B, C**). Previous studies have confirmed their roles in regulating genes involved in drought, salinity, and cold stress responses (Lorenzo et al., 2004; Tohge et al., 2005; Xiong et al., 2014). These findings suggest that *CiGRF* genes are likely regulated at the transcriptional level via stress-related CREs and thus may play critical roles in environmental stress adaptation.

Similar to *CiGRF* promoters, GRF promoters from *C. makinoi*, *C. nankingense*, and *C. lavandulifolium* also contained abundant stress- and hormone-responsive elements, including MYB, MYC, LTR, W-box, and methyl jasmonate (MeJA) - responsive motifs (**Supplementary Figure S5**). This overall conservation indicates that GRF genes across *Chrysanthemum* species share a common stress-responsive regulatory framework. However, differences in CREs composition were observed. Notably, the abscisic acid-responsive element ABRE was detected exclusively in the GRF promoters of *C. makinoi*, and these promoters also exhibited a relatively higher proportion of CGTCA motifs, which are associated with MeJA responsiveness. This pattern suggests that transcriptional regulation of GRF genes in *C. makinoi* may rely more strongly on hormone-mediated signaling pathways. In contrast, the promoters of *C. nankingense* GRFs were predominantly enriched with stress-responsive cis-elements, while hormone-related elements such as ABRE were less frequent, indicating a stress-signal-dominated regulatory pattern. These differences suggest that, while GRF genes are broadly conserved, their transcriptional regulation may rely on distinct upstream signaling inputs among *Chrysanthemum* species, potentially reflecting species-specific regulatory strategies for integrating stress and hormonal signals.

Building upon its documented resilience and wide ecological distribution, our CREs analysis revealed that *C. indicum* GRF genes possess the most enriched and complex repertoire of stress-responsive cis-elements among the studied *Chrysanthemum* species. This molecular characteristic suggests a potent and sophisticated transcriptional regulatory potential under stress. Therefore, to functionally validate the link between these distinctive cis-regulatory architectures and stress responses, *C. indicum* was selected as the representative model for subsequent expression profiling of GRF genes under various abiotic stresses.

### 3.6 Expression analysis of GRF genes under abiotic stress

**Figure 7:**
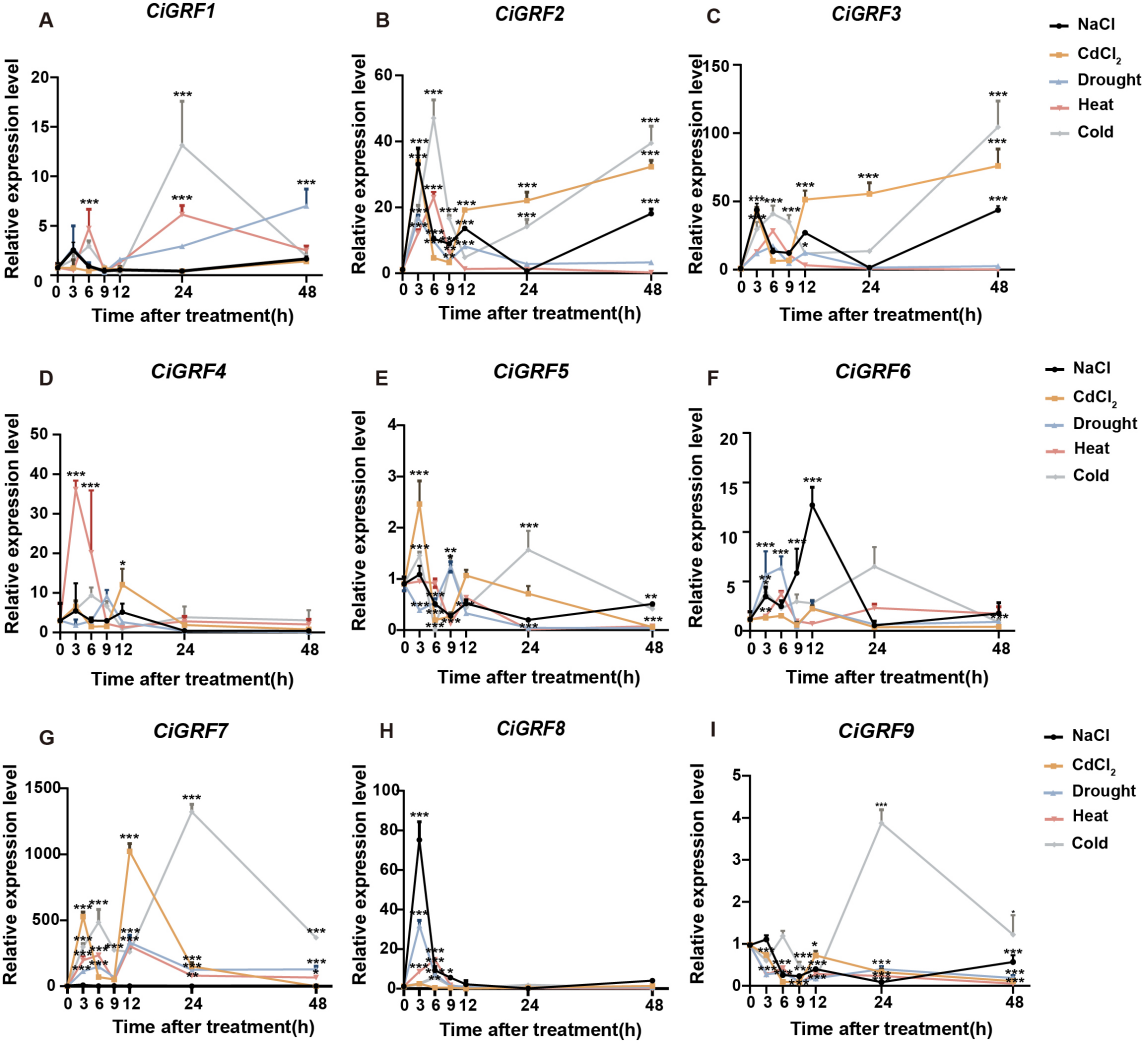
Expression analysis of *CiGRF* genes under abiotic stresses. **(A-I)** qRT-PCR analysis of *CiGRF* expression under NaCl, CdCl_2_, drought, cold, and heat treatments. Relative expression levels were calculated via 2^-ΔΔCT^ method. Data are presented as mean ± s.d. (n = 3). Asterisks denote significant differences by Student’s *t*-test (*P < 0.05; **P < 0.01; ***P < 0.001).

Consistent with promoter analysis results, *CiGRF* promoters were found to be rich in stress-responsive elements, indicating potential roles in abiotic stress responses. Thus, we analyzed the expression of nine *CiGRFs* under five stresses: NaCl, CdCl_2_, drought, cold, and heat (**Figure 7**). All nine *CiGRFs* showed varying degrees of upregulation under at least one stress condition. *CiGRF6* and *CiGRF8* responded strongly to NaCl treatment, with expression levels increasing several dozen - fold, indicating high salt sensitivity (**Figures 7F, H**). *CiGRF7* exhibited a rapid response to CdCl_2_ stress, peaking at 3 h post-treatment. Under CdCl_2_, drought, heat, and cold stress, most genes exhibited biphasic expression peaks within 24 h, suggesting a coordinated stress response pattern.

### 3.7 Subcellular localization and transcriptional activation of *CiGRF7*

**Figure 8:**
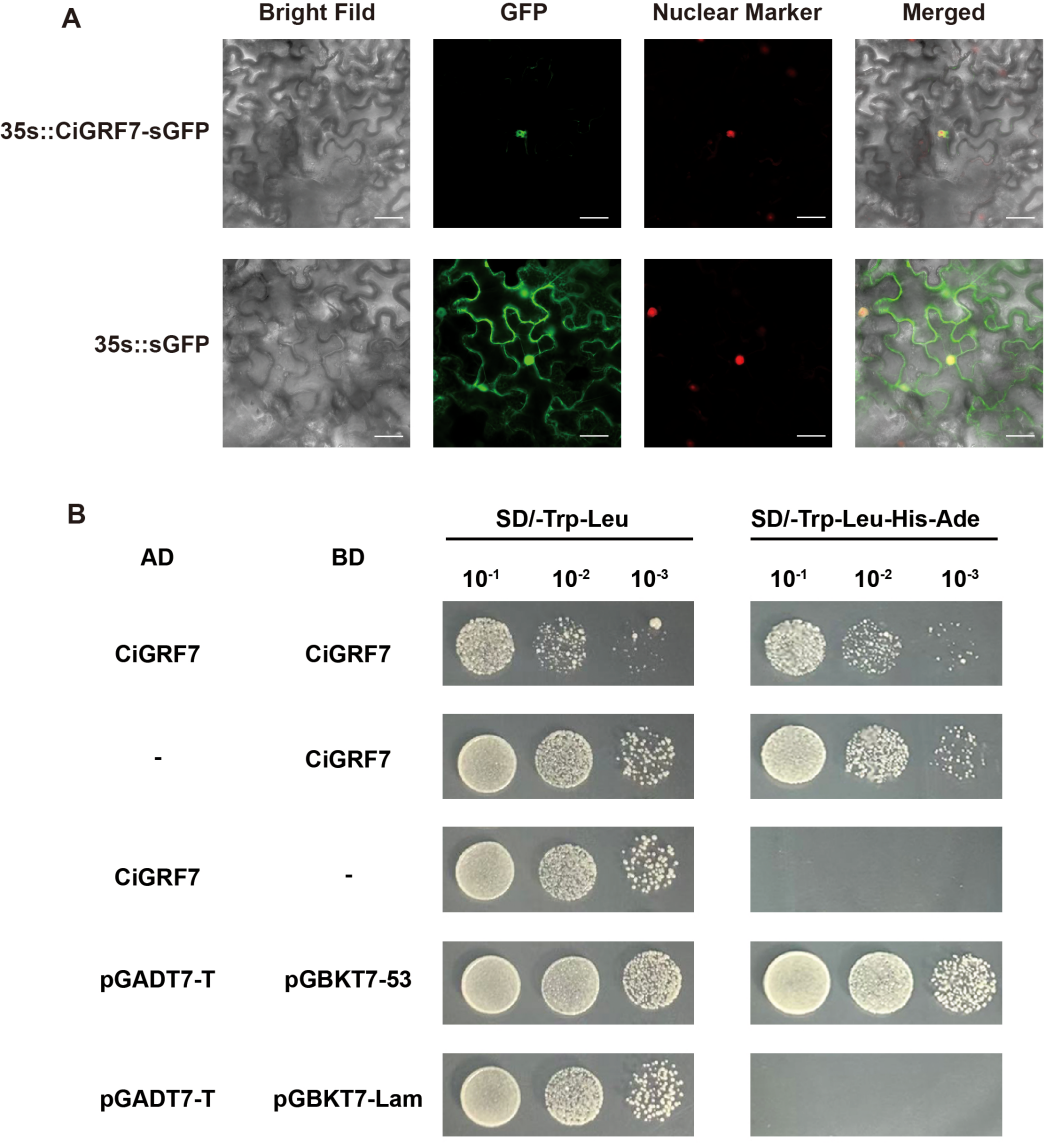
Subcellular localization and transcriptional activation of CiGRF7 protein. **(A)** Localization of CiGRF7 - GFP fusion protein in *Nicotiana benthamiana* leaves. D53-mCherry served as nuclear marker. Scale bars: 5 μm. **(B)** Transcriptional self-activation assay of CiGRF7 in yeast. pGADT7-T + pGBKT7–53 and pGADT7-T + pGBKT7-Lam were used as positive and negative control, respectively.

Previous expression profiling suggested that *CiGRF7* is a key candidate involved in cold stress responses. To further explore its molecular characteristics, we performed subcellular localization and transcriptional activation assays. Sequence analysis revealed that *CiGRF7* contains a canonical nuclear localization signals (NLS) (**Supplementary Figure S6**). Its coding sequence was cloned into the 35S::*CiGRF*-sGFP construct and transiently expressed in *Nicotiana benthamiana* leaves. Confocal microscopy showed that GFP fluorescence signals colocalized with mCherry nuclear marker (**Figure 8A**), confirming nuclear localization.

Transcriptional activation assays in yeast demonstrated that CiGRF7 possess self-activating capabilities and can form dimers, further supporting its role as a transcriptional activator (**Figure 8B**). These findings suggest that CiGRF7 functions as a nuclear-localized transcription activator involved in the regulation of abiotic stress responses in *Chrysanthemum*.

## 4 Discussion

*Chrysanthemum* is a major ornamental and medicinal plant in China with high aesthetic/commercial value. Comprehensive understanding of its growth and stress adaption mechanisms is essential for sustainable development (Li et al., 2023; Wang et al., 2023b). GRF genes, plant-specific transcription factors, play crucial roles in regulating growth, development, and environmental responses (Kim and Lee, 2006; Omidbakhshfard et al., 2015, 2018; Liebsch and Palatnik, 2020). Compared to algae/bryophytes (typically 1–2 GRFs), higher plants harbor 8–20 GRFs (Kim et al., 2003; Choi et al., 2004; Jin et al., 2014; Omidbakhshfard et al., 2015). Despite their significance, GRFs in *Chrysanthemum* species have remained poorly characterized.

Here, we identified a total of 43 GRF genes across four *Chrysanthemum* species: *C. indicum* (9), *C. makinoi* (11), *C. nankingense* (11), and *C. lavandulifolium* (12) (**Figure 1**, **Supplementary Table S2**). These numbers are comparable to those in model plants such as *Arabidopsis thaliana*, *Oryza sativa*, and *Zea mays* (Kim et al., 2003; Choi et al., 2004; Zhang et al., 2008), indicating evolutionary stability. Phylogenetic analyses revealed that GRF expansion is largely driven by whole-genome duplication (WGD) and segmental duplication (**Figure 4**). Collinearity showed the strongest synteny between *C. indicum* and *C. nankingense* (**Figure 3**), supporting the classification of the latter as a *C. indicum* variety (Zhong et al., 2024).

Structural analysis revealed that most *Chrysanthemum* GRF genes contain 3–4 exons, and the conserved motifs motif1 and motif2 were highly conserved across members (**Figure 2B**). This structural conservation is consistent with findings in other species such as rice, tobacco, cabbage, and maize (Wang et al., 2014a, 2023a; Zhang et al., 2018b; Qin et al., 2022), suggesting a conserved functional framework for GRFs across diverse plant lineages. GRF biological functions are well-characterized in model plants (*Arabidopsis*, rice, maize, wheat) (Kim et al., 2012; Wu et al., 2014; Li et al., 2016, 2019; Omidbakhshfard et al., 2018; Tang et al., 2018; Zhang et al., 2018a). With expanding medicinal plant genomes (Meng et al., 2022), *Chrysanthemum* GRF research - given its dual ornamental - medicinal value - grows increasingly relevant. Spatiotemporal analysis showed that GRFs are expressed across organs (roots, stems, buds, leaves) and floral stages (ray/disc florets) (**Figure 5**), indicating roles in multiple developmental processes. Promoter analysis further showed enrichment of stress - responsive, growth - related, and hormonal signaling CREs (Dai et al., 2019; Hu et al., 2023; Yuan et al., 2024a, 2024b; Nazir et al., 2025). Notably, MYB/MYC - binding sites dominated promoter regions (**Figures 6B, C**), indicating GRFs function critically in abiotic stress adaptation.

Abiotic stresses including heat, drought, and salinity impair *Chrysanthemum* growth, bioactive accumulation, and economic value (Wang et al., 2022; Xia et al., 2025; Luo et al., n.d.). Here, GRF genes showed diverse expression responses to NaCl, drought, heat, cold, and CdCl_2_ (**Figure 7**). Notably, *CiGRF1*, *CiGRF2*, *CiGRF4*, *CiGRF6*, and *CiGRF7* were significantly upregulated under multiple stresses (**Figures 7A, B, D, F, G**). *CiGRF7* exhibited >1000-fold induction under cold/CdCl_2_ stress (**Figure 7G**), indicating its central role in cold/ion responses. Conserved domain analysis revealed that *CiGRF7* shares highly conserved WRC and QLQ domains with orthologs like rice *OsGRF4* (**Supplementary Figure S7**). Given *OsGRF4*’s roles in stress responses and floral development (Li et al., 2018; Shen et al., 2024), *CiGRF7* may similarly regulate floral organ development and environmental adaptation.

To further explore the regulatory characteristics of *CiGRF7*, we examined its transcriptional activation capacity. Subcellular localization and yeast assays demonstrated that *CiGRF7* is a nuclear-localized transcriptional activator (**Figure 8**). From a structural perspective, GRF proteins generally possess two conserved N-terminal domains, WRC and QLQ, which are primarily responsible for DNA binding and protein-protein interactions rather than transcriptional activation. Previous studies in *Arabidopsis*, rice, and maize have shown that GRF activation domains are typically located within the non-conserved, low-complexity C-terminal region (Choi et al., 2004). Consistent with this pattern, our motif analyses showed that the C terminus of *CiGRF7* is highly divergent and lacks conserved motifs, matching the common characteristics of activation domains. Similar pattern is also found in grape GRFs, where only *VvGRF8* and *VvGRF9*-both possessing long and low-complexity C-terminal regions-exhibited strong transactivation activity, whereas proteins with truncated C termini showed weak or GIF-dependent activation (Hu et al., 2023). Together, these results not only support the presence of transcriptional activity in *CiGRF7* but also provide strong evidence that its activation domain is likely located within the C-terminal variable region. Precise delineation of the minimal activation fragment will require systematic N- and C-terminal truncation analyses, which will be addressed in future studies to further refine the mechanistic understanding of *CiGRF7*-mediated transcriptional regulation.

Although this study provides functional insights into the transcriptional properties and stress-responsive expression of CiGRF7, its precise biological role in regulating abiotic stress tolerance *in vivo* remains to be fully elucidated. Future studies employing genetic approaches, including overexpression, CRISPR/Cas9-mediated knockout, and complementation analyses, will be essential to definitively clarify the physiological functions and regulatory mechanisms of CiGRF7 under stress conditions.

Beyond *CiGRF7*, the downstream targets and interaction networks of GRF family members in *Chrysanthemum* remain largely unknown. Future research should therefore focus on integrating genetic, transcriptomic, and protein-protein interaction analyses to comprehensively elucidate GRF-mediated regulatory networks involved in growth control and environmental adaptation.

Collectively, these findings deepen our understanding of GRF gene evolution and stress-responsive regulation in *Chrysanthemum* and provide valuable genetic resources for breeding programs aimed at improving stress tolerance, growth performance, and ornamental and medicinal quality.

## 5 Conclusions

In this study, 43 GRF genes were identified from four *Chrysanthemum* species and classified into four clades with conserved structures, motifs, and evolutionary patterns. Expression profiling revealed distinct tissue- and stage-specific patterns, while promoter analysis showed enrichment of stress-responsive cis-elements. qRT-PCR confirmed that several *CiGRFs*, especially *CiGRF7*, were strongly induced by salinity, drought, extreme temperatures, and CdCl_2_, with *CiGRF7* exhibiting over 1,000-fold upregulation under cold and heavy metal stress. Subcellular localization and transcriptional activation assays indicated that *CiGRF7* acts as nuclear-localized transcriptional activators. These results provide insights into GRF gene functions and valuable resources for improving stress tolerance and development in *Chrysanthemum*.

## Data Availability

The original contributions presented in the study are included in the article/**Supplementary Material**. Further inquiries can be directed to the corresponding authors.
