# Supplementary material for: Identification and functional analysis of growth- regulating factors involved in abiotic stress response in Chrysanthemum L
Source: Front Plant Sci. 2026 Jan 20;16:1744200. doi: 10.3389/fpls.2025.1744200 (PMC12864121; doi:10.3389/fpls.2025.1744200)
Supplement: Supplementary file 1 [file SupplementaryFile1.docx]

Supplementary Material

# Supplementary Figures


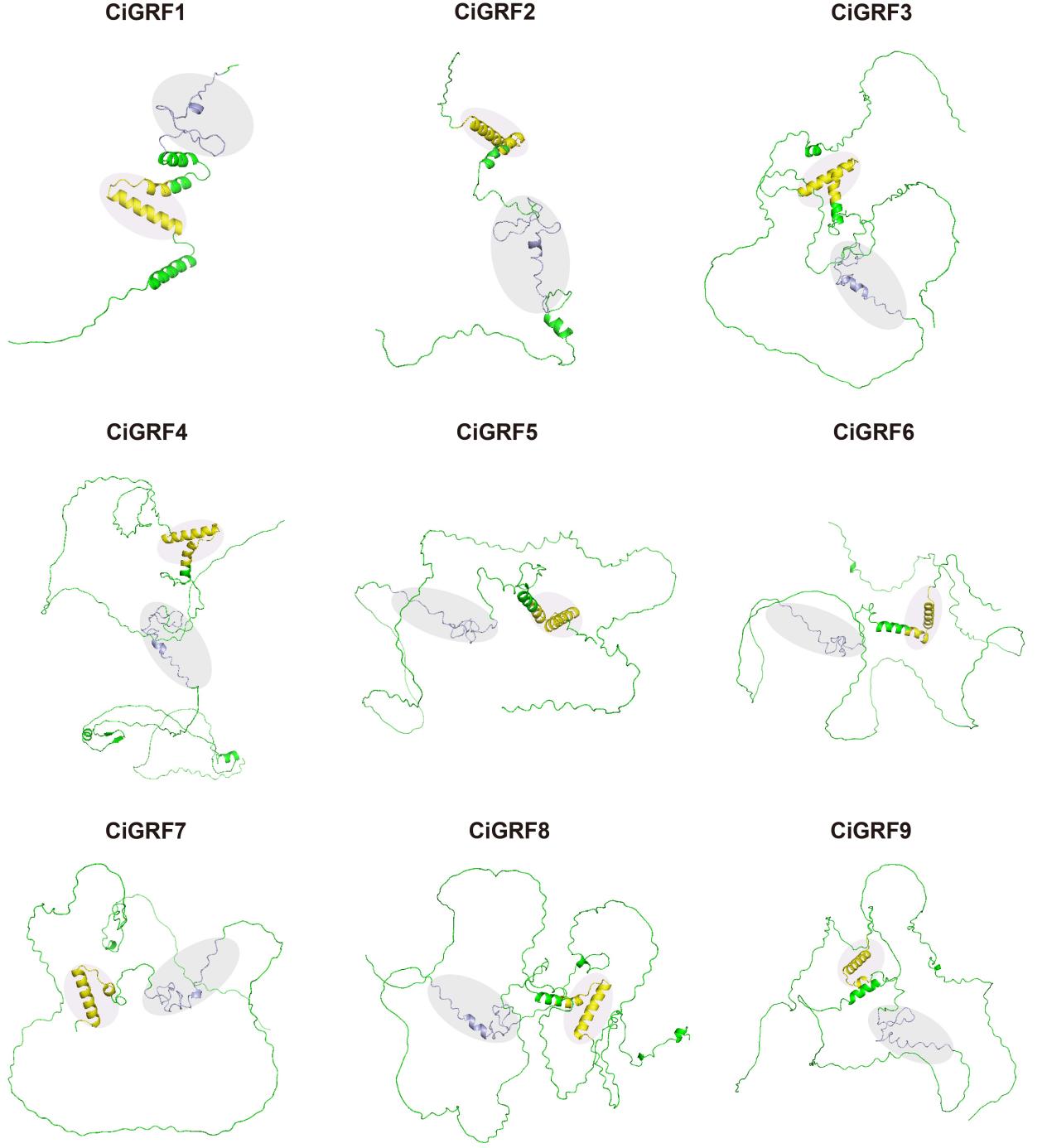


**Supplementary Figure 1.** Three-dimensional structural analysis of GRF proteins in *C. indicum*. The conserved QLQ and WRC domains are highlighted in pink and purple, respectively.


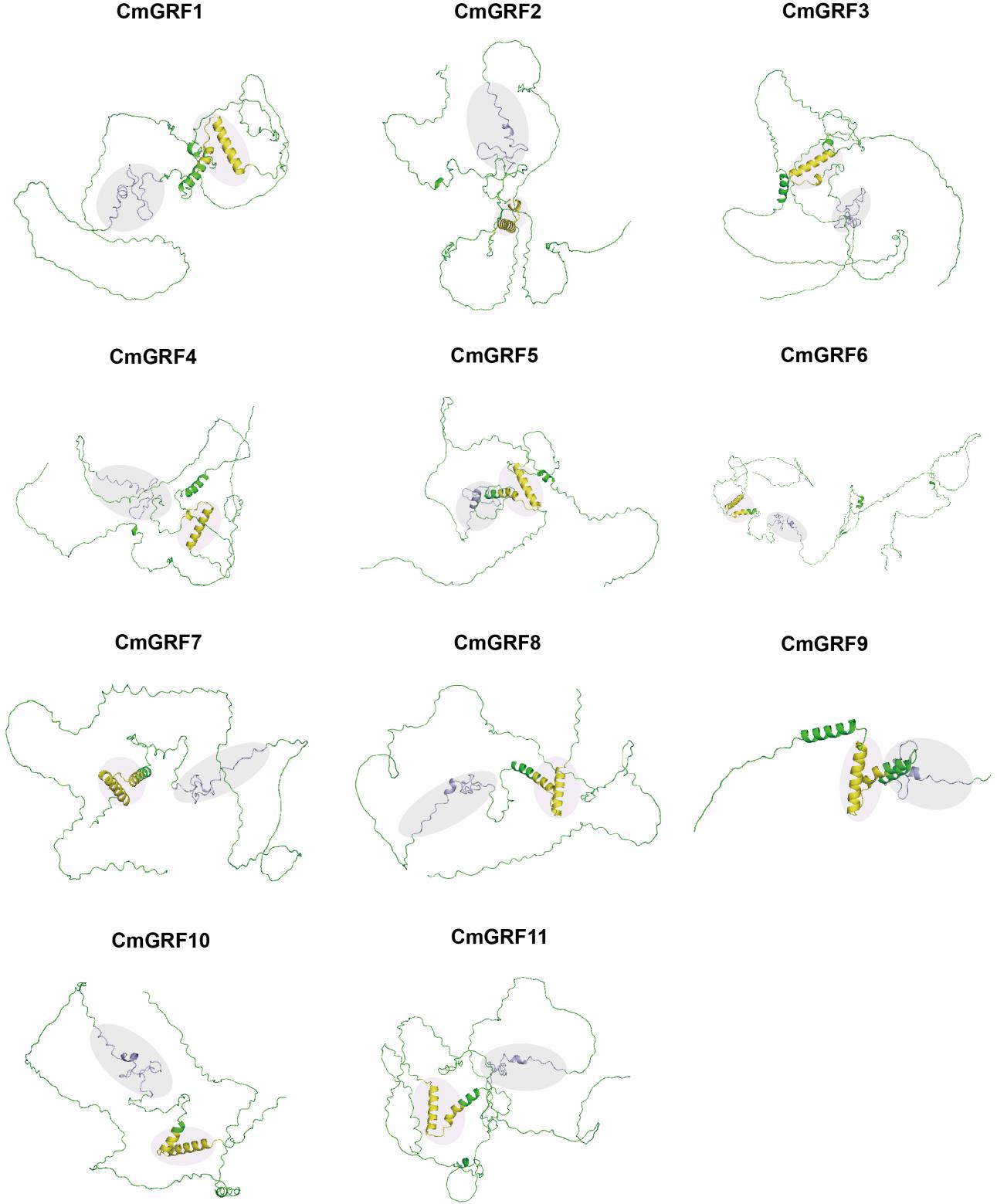


**Supplementary Figure 2.** Three-dimensional structural analysis of GRF proteins in *C. makinoi*. The conserved QLQ and WRC domains are highlighted in pink and purple, respectively.


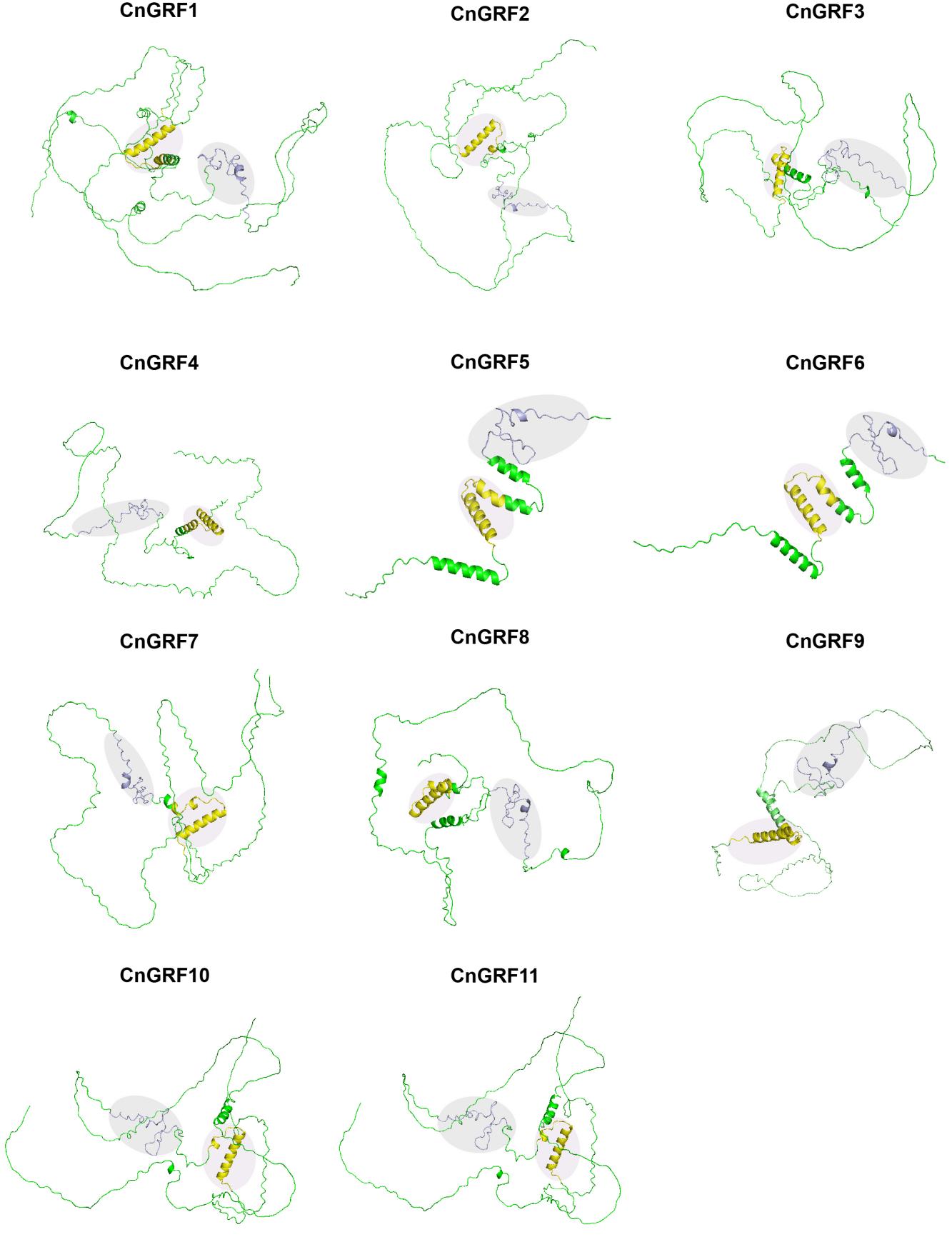


**Supplementary Figure 3.** Three-dimensional structural analysis of GRF proteins in *C. nankingense.* The conserved QLQ and WRC domains are highlighted in pink and purple, respectively.

**
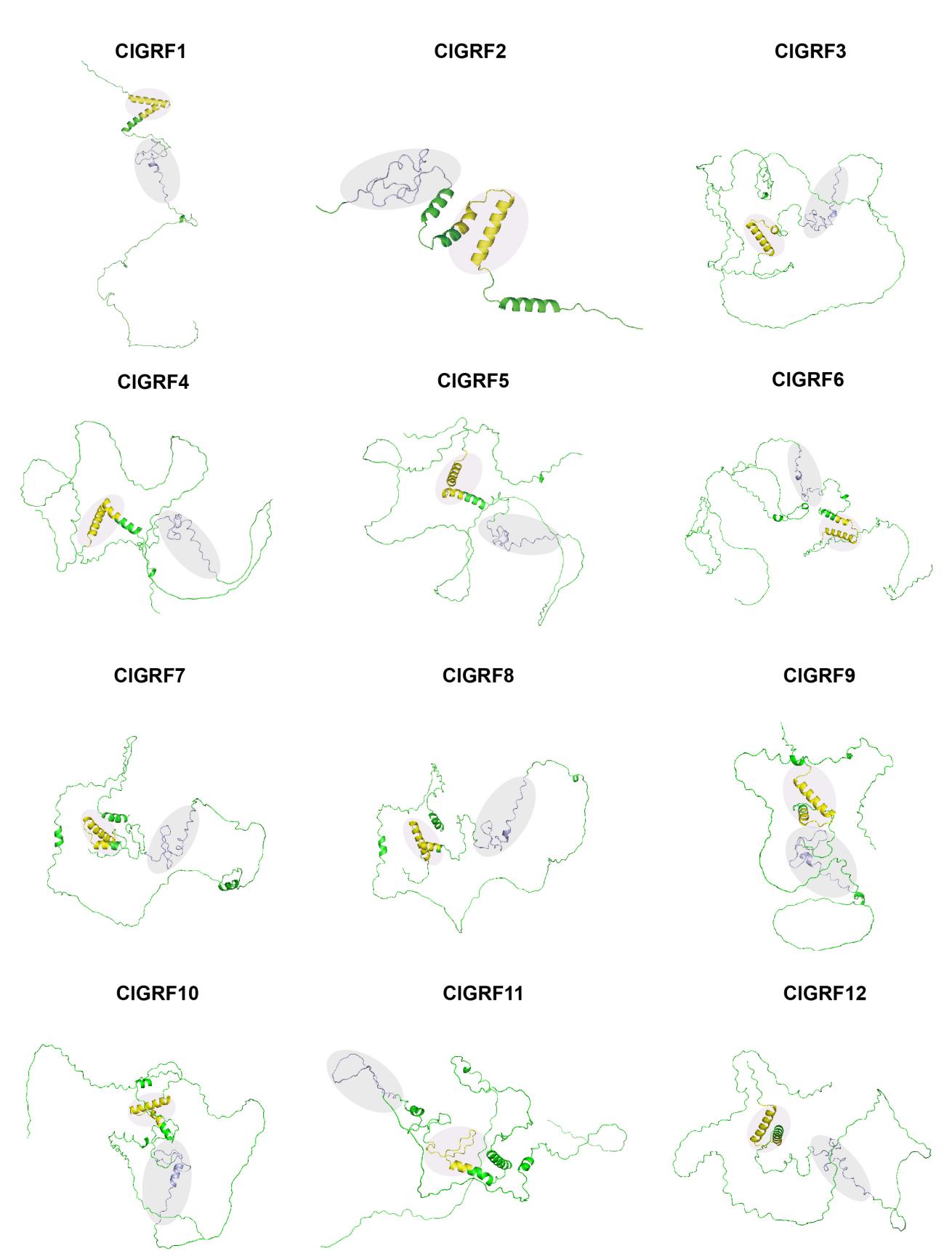
**

**Supplementary Figure 4.** Three-dimensional structural analysis of GRF proteins in *C. lavandulifolium*. The conserved QLQ and WRC domains are highlighted in pink and purple, respectively.


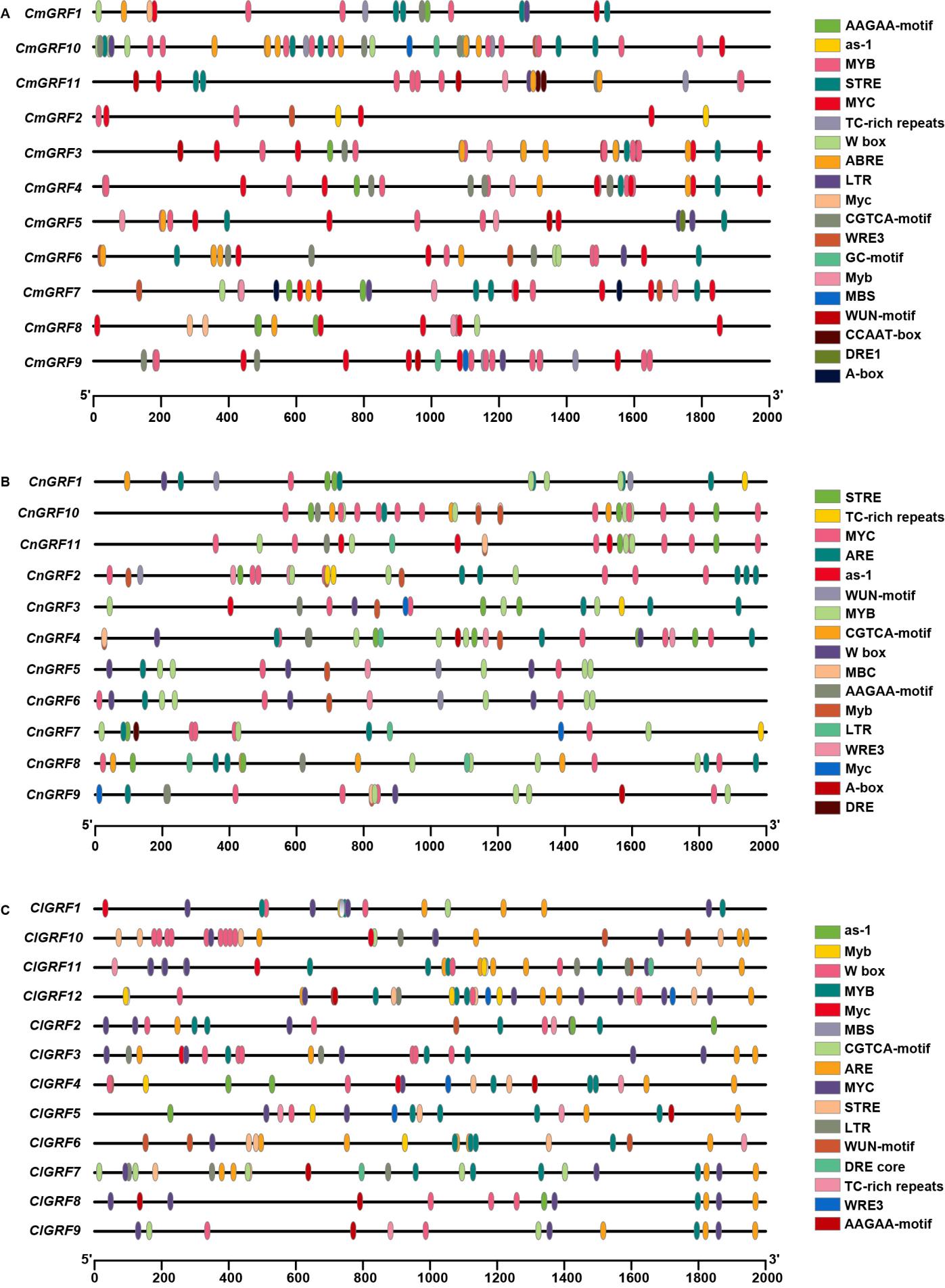


**Supplementary Figure 5**. Distribution of major stress-related CREs in the promoter sequences of the GRF genes from (A) *C. makinoi*, (B) *C. nankingense,* and (C) *C.* *lavandulifolium*.


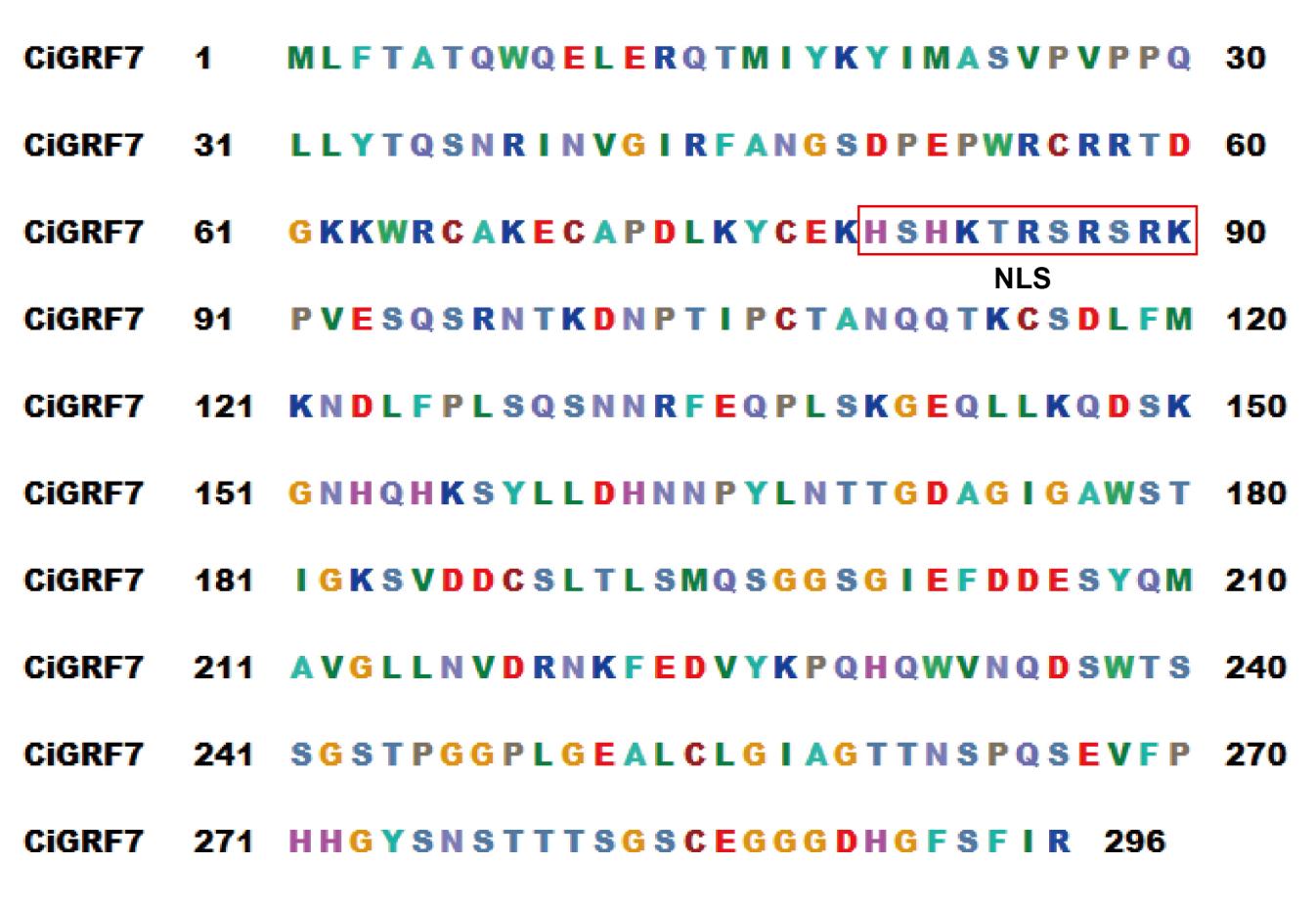


**Supplementary Figure 6.** The prediction of nuclear localization signal was performed using the NLStradamus website. The red box indicates the predicted nuclear localization signal.

**
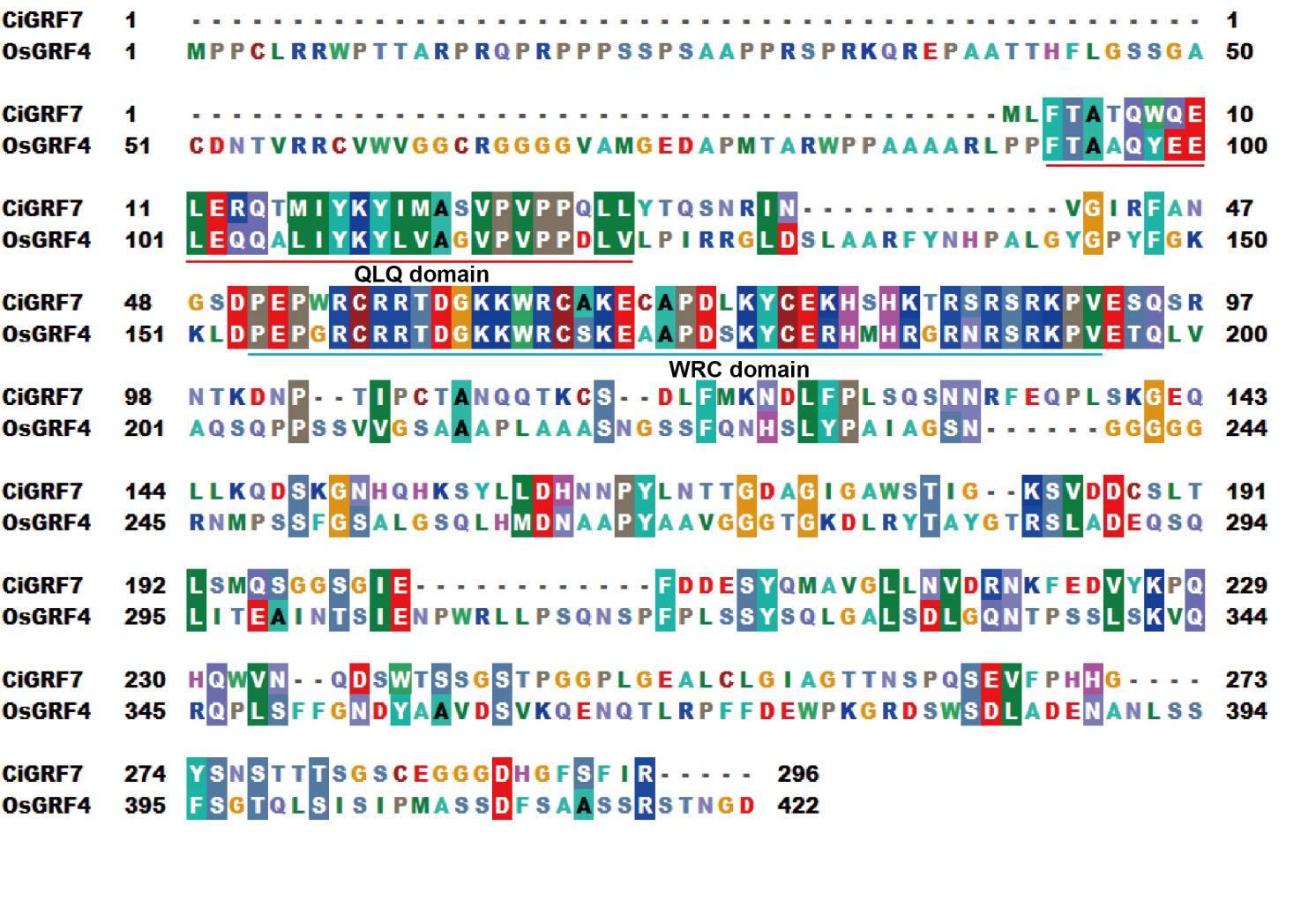
**

**Supplementary Figure 7.** Protein sequence difference analysis between CiGRF7 and its rice homolog OsGRF4. The CiGRF7 sequence was aligned using BioEdit. The conserved QLQ and WRC domains are indicated by red and blue lines, respectively.
